# Polyetheretherketone development in bone tissue engineering and orthopedic surgery

**DOI:** 10.3389/fbioe.2023.1207277

**Published:** 2023-06-29

**Authors:** Zhanqi Wei, Ze Zhang, Wei Zhu, Xisheng Weng

**Affiliations:** ^1^ Department of Orthopedics, Peking Union Medical College Hospital, Chinese Academy of Medical Sciences and Peking Union Medical College, Beijing, China; ^2^ School of Medicine, Tsinghua University, Beijing, China; ^3^ Department of State Key Laboratory of Complex Severe and Rare Diseases, Peking Union Medical College Hospital, Chinese Academy of Medical Science and Peking Union Medical College, Beijing, China

**Keywords:** polyetheretherketone, osseointegration, antibacterial property, spine surgery, joint surgery, trauma repair

## Abstract

Polyetheretherketone (PEEK) has been widely used in the medical field as an implant material, especially in bone tissue engineering and orthopedic surgery, in recent years. This material exhibits superior stability at high temperatures and is biosecured without harmful reactions. However, the chemical and biological inertness of PEEK still limits its applications. Recently, many approaches have been applied to improve its performance, including the modulation of physical morphology, chemical composition and antimicrobial agents, which advanced the osteointegration as well as antibacterial properties of PEEK materials. Based on the evolution of PEEK biomedical devices, many studies on the use of PEEK implants in spine surgery, joint surgery and trauma repair have been performed in the past few years, in most of which PEEK implants show better outcomes than traditional metal implants. This paper summarizes recent studies on the modification and application of biomedical PEEK materials, which provides further research directions for PEEK implants.

## 1 Introduction

Polyetheretherketone (PEEK), a semicrystalline thermoplastic polymer with a chemical formula of (–C_6_H_4_–CO–C_6_H_4_–O–C_6_H_4_–O–)_n_, is formed by polymerizing ether ketone monomer units via a stepwise growth dialkylation reaction of bis-phenolates (Najeeb et al., 2016). PEEK exhibits excellent physicochemical properties; it has a high melting temperature of 334°C, so its chemical structure remains stable at temperatures exceeding 300°C; it resists harsh chemicals, such as organic solvents, acids and bases; it exhibits a high strength and good fatigue and abrasion resistance; and it has good compatibility with many reinforcing agents (Stolarski, 1992; Ponnappan et al., 2009).

When materials are used in living organisms as biomedical materials, it is crucial to evaluate their biocompatibility and stability. As a bioinert material, PEEK does not exhibit any mutagenic or cytotoxic activity and does not produce any harmful reactions or release any harmful components into human tissues (Verma et al., 2021). Compared with titanium and other traditional metal alloys, PEEK has a low elastic modulus (3-4 GPa), which is near that of human cortical bone (7–30 GPa), reducing the extent of stress shielding (Kizuki et al., 2015; Panayotov et al., 2016). In addition, PEEK can be easily molded into bone shapes by multiple sterilization and thermal contouring with conventional plastic processing equipment, which facilitates its production and application (Wang et al., 2014). Comparing with metal implants, PEEK implants also display satisfied X-ray permeability allowing the evaluation of adjacent anatomic structures during radiography, computed tomography (CT), and magnetic resonance imaging (MRI) (Long et al., 2023).

According to above advantages, various modified PEEK composites have been widely applied in bone tissue engineering and orthopedic surgeries, such as:

(1) Used as spinal fusion cages or rods in lumbar and cervical spine surgeries;
(2) Used as suture anchors, joint replacements or plates in joint surgeries;
(3) Used as reconstructive implants or intramedullary nails in cranioplasty and fracture fixation.

Despite these advantages and broad application areas, the biological inertness and weak antibiological ability of PEEK leading to unsatisfied results in some aspects. The lack of osteointegration and antibacterial properties may cause the aseptic loosening, infectious complications, early revision events, and even the implant failure. It is reported that 15.3% developed post-operative complications and 8.7% experienced implant failure requiring reoperation of 183 PEEK cranioplasty patients (Punchak et al., 2017). The existing defects have negative impact on using PEEK materials as implant materials in biomedical field. Therefore, the ideal PEEK implants should maintain the existing physicochemical properties, biocompatibility and stability, and the osteointegration as well as antibacterial properties need to be improved. Thus, many studies have been carried out in recent years to improve cell attachment, cell proliferation, mechanical strength as well as antibacterial property of PEEK by modifying physical morphology, adding chemical composition, introducing antibiotics and so on.

Herein, in this review, we summarize the up-to-date strategies used for improving osteointegration and antibacterial properties of PEEK and the current clinical studies about the application of PEEK in bone tissue engineering and orthopedics.

## 2 Strategies for improving osteointegration

The stable binding of implants with bone is an important point for evaluation the operative outcomes. After the implant surgeries, activated blood cells release differentiation and growth factors at the interface, which promote the bone formation around the implants, and the implant interface is filled with bone ultimately (Apostu et al., 2018). This process is called osteointegration, which is a key process to keep the implant surviving and stable. While artificial implants without sufficient osteoconductivity or osteoinductivity will lead to the failure of osteointegration. After the implantation, macrophages aggregate to biomaterials-tissue interface and form multinucleated foreign body giant cells, and then activate the fibroblast which secrets collagen fibers forming a fibrous capsule on surface of biomaterials. The fibrous encapsulation separates the implant with tissue resulting in insufficient mechanical fixation (Liu et al., 2021). This process causes the aseptic loosening, premature failure of implants and early revision events. Excellent chemical resistance is accompanied by biological inertness, which leads to the inferior osteogenesis and osseointegration caused by PEEK (Toth et al., 2006). Many approaches, such as surface physical treatments (e.g., sandblasting, magnetron sputtering and plasma treatment) (Ourahmoune et al., 2014; Poulsson et al., 2014; Hussain et al., 2020), chemical modifications (e.g., fluoridation, sulfonation and nitrification treatment, and functional group grafting) (Chen M. et al., 2017; Li et al., 2019), and coating with one-layer or layer-by-layer (LbL) bioactive materials (e.g., hydroxyapatite) (Barkarmo et al., 2013), have been developed to serve to regulate the physical (e.g., morphology) and chemical (e.g., composition) properties of PEEK and thus the osteogenic activity of the PEEK implant surface.

### 2.1 Modulation of physical morphology

Physical morphology regulation plays a crucial role in controlling cell adhesion and differentiation by affecting cell-generated physical forces, thus improving the osteointegration of the substrate. Surface topography is a typical physical topography that can affect osteogenic nanomorphology by reorganizing cytoskeletal ultrastructure and morphology, directing cell adhesion, and influencing cell signaling and metabolism (Dalby et al., 2014). Because elemental doping modifications may be toxic to cells, blood and organs, biomaterials with surface topography modifications are more promising for clinical applications due to their higher safety. In addition, implants with rough morphologies are more conducive to initial cell fixation than smooth structured implants, thereby enhancing osteoblast attachment and differentiation (Kang et al., 2015; Baino et al., 2016).

#### 2.1.1 Modification of physical parameters

Parameters including the implant pore size, porous structure, and morphology have impacts on bioactivity. To explore the optimum pore size, Feng et al. (Feng et al., 2020) manufactured PEEK scaffolds with various pore sizes and a porosity of 60% via fused deposition modeling (FDM) and reported that PEEK scaffolds with 450 μm pore sizes were most beneficial for cell adhesion, proliferation, and osteogenic differentiation and exhibited preferential potential for bone ingrowth and vascular perfusion. The synergy of different pore sizes also contributes to better performance. Wang et al. (Wang L. et al., 2019) revealed that the porosity, water absorption, and protein absorption of the porous surface of a PEEK-nanoporous lithium-doped magnesium silicate (NLS) blend (PKNLS) with macropores and nanopores were obviously enhanced compared to those of PKLS and PEEK with macropores but without nanopores, and the porous structure exhibited more influence on mechanical properties than the pore size.

#### 2.1.2 Modification of surface morphology

To obtain PEEK with a surface structure with good osteogenic capacity, the high temperature or active particles of plasma were used to chemically treat a material surface. The treatment of metal or nonmetallic solid surfaces with cold plasma can also play a mechanical processing role. For example, Ouyang et al. (Ouyang et al., 2019) constructed a nanostructure on the PEEK surface by argon plasma immersion ion implantation (PIII) and subsequent hydrogen peroxide treatment and reported that the surface fabricated by physical-chemical treatment was more favorable for fibrous tissue filtration inhibition and osseointegration than that fabricated by argon PIII only. This work provides a potential approach for improving the osseointegration ability of PEEK implants by nanostructural surface modification.

Unlike the traditional processing mode of cutting and assembling raw materials, additive manufacturing (AM), commonly known as three-dimensional (3D) printing, is a “bottom-up” manufacturing method that makes it possible to manufacture complex structural parts that were not possible to manufacture in the past due to the constraints of traditional manufacturing methods (Gross et al., 2014; Najeeb et al., 2015). 3D printing PEEK to fabricate orthopedic implants has been applied in a fairly wide range of applications (Singh et al., 2019). Oladapo et al. (Oladapo et al., 2020) fabricated a PEEK-calcium hydroxyapatite (cHAp) biocomposite with microporous architectures by the FDM method. The porous nanostructure of PEEK has a controlled pore size and distribution to promote cell penetration and the biological integration of PEEK-cHAp into tissue. *In vivo* tests demonstrated that the surface-treated micropores facilitated the adhesion of newly regenerated soft tissues to form tight implant-tissue interfacial bonding between cHAp and PEEK.

**FIGURE 1:**
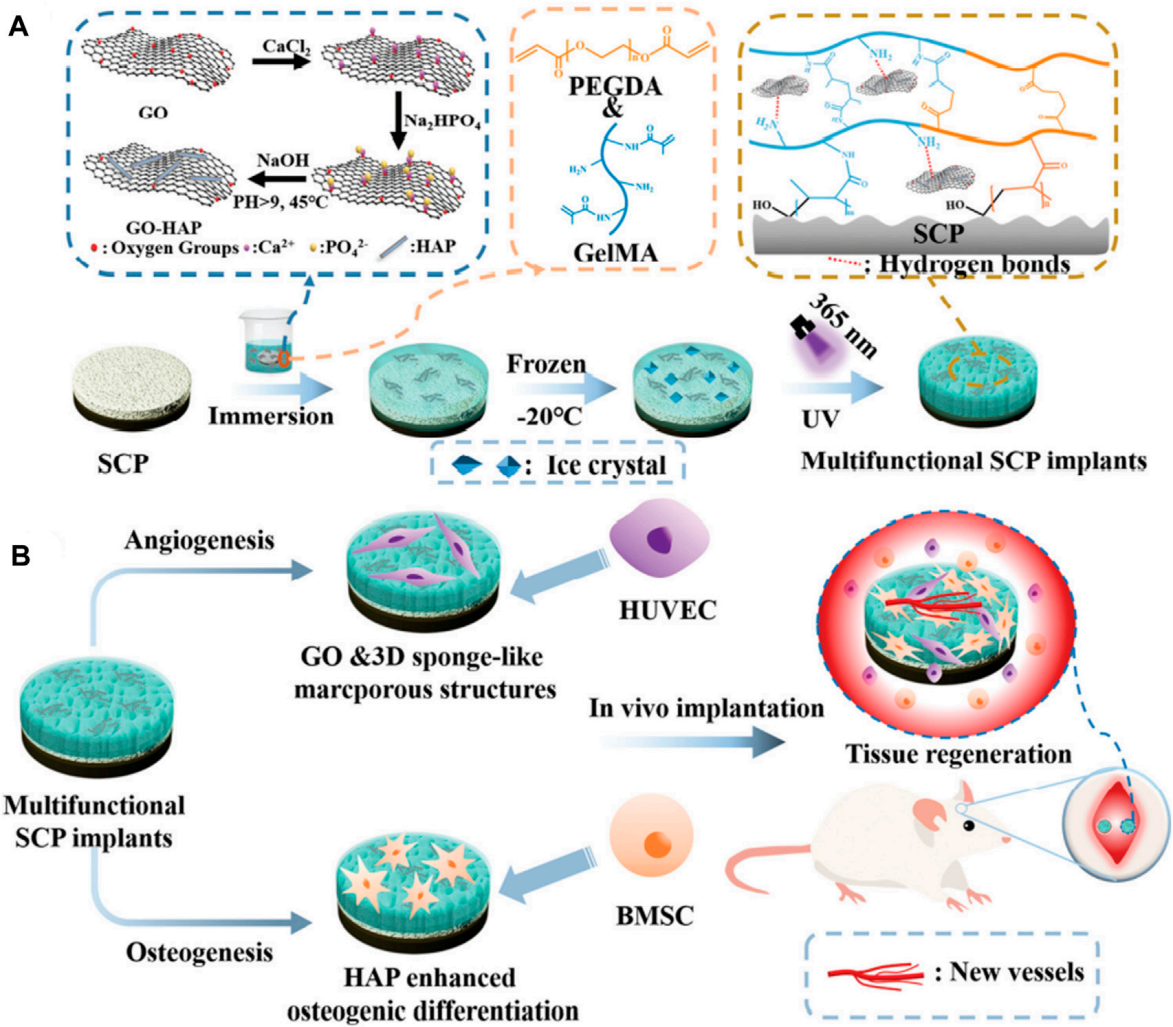
The modification procedures on the sulfonated LCFRPEEK surface and its biological functions: angiogenesis and osteogenesis. Reprinted with permission from Ref. (Gross et al., 2014). 2022, The Royal Society of Chemistry.

Free radical photopolymerization, which uses ultraviolet light to destroy the diphenyl ketone units of PEEK implants and graft the reactive monomers or polymers onto the implant surface to form a three-dimensional gel network, is also used for morphology modification (Yousaf et al., 2014). PEEK materials typically exhibit less strength than human bone, and their porous structures generally weaken their mechanical properties as well. Long carbon fiber-reinforced polyetheretherketone (LCFRPEEK) composites exhibit an elastic modulus (18–35 GPa) comparable to that of human cortical bone but lack osteogenesis abilities due to their bioinert surface. Dong et al. (Dong et al., 2022) fabricated a multifunctional 3D sponge-like macroporous cryogel on an inert sulfonated LCFRPEEK implant. The addition of graphene oxide (GO) and the construction of 3D macroporous structures can synergistically promote *in situ* osseointegration and angiogenesis (Figure 1).

It is a good solution to build surface nanostructures on PEEK to improve their osteogenic activity and maintain their excellent properties. Zhang et al. (Zhang et al., 2022) developed a facile hot die formation technique for modifying PEEK material surfaces with *in situ* patterned nanorod arrays. The results revealed that surface structure-engineered PEEK induced the differentiation of stem cells into osteoblasts in the absence of biological or organic small-molecule differentiation-inducing factors. The subcutaneous implantation and bone defect repair models showed that the implants with patterned nanorod arrays exhibited improved osseointegration properties.

### 2.2 Modulation of chemical composition

Compared to physical morphology modifications, chemical composition modifications allow greater performance optimization due to the introduction of new components. Common chemical composition modulation methods include element doping, grafting functional groups and organic/inorganic coatings.

#### 2.2.1 Element doping

Element doping is an effective way to functionalize PEEK materials. A variety of metal ions, including copper, silver, titanium, strontium and zinc ions, are added to PEEK implants to regulate polarization and activate macrophage pathways, thereby regulating the inflammatory response and improving the osteogenic function of PEEK implants (Han et al., 2010; Zhang et al., 2016; Liu et al., 2018; Liu et al., 2019; Xu et al., 2019). Cytokines released by immune cells are important in inducing bone tissue regeneration, and the biomaterial microenvironment can influence the production of these bone-targeting macrophage cytokines. Liu et al. (Liu et al., 2018) revealed that the microenvironment of Zn-doped sulfonated polyetheretherketone (SPEEK) modulated nonactivated macrophage polarization into an anti-inflammatory phenotype and induced the secretion of anti-inflammatory and osteogenic cytokines. The osteogenic differentiation capability of bone marrow stromal cells (BMSCs) was therefore enhanced, leading to improved osteointegration between the zinc-doped SPEEK and bone tissue. Similarly, copper, magnesium, strontium and titanium ions have been verified to modulate the macrophage phenotype and improve implant osseointegration.

Nonmetallic elements also play an important role in improving the osteogenic function of PEEK implants. Trace fluorine can promote osteoblast proliferation, upregulate alkaline phosphatase (ALP) activity, and stimulate osteocalcin and collagen I synthesis (Farley et al., 1983). Through argon PIII followed by hydrofluoric acid treatment, Chen et al. (Chen M. et al., 2017) prepared fluorinated PEEK, which enhanced cell adhesion, spreading and proliferation and ALP activity compared to pristine PEEK and showed superior osseointegration and bacteriostasis properties.

#### 2.2.2 Inorganic composition coating

Besides the element doping, various inorganic bioactive materials including metallic and nonmetallic materials have been used as coatings to improve the cell affinity and osteointegration of PEEK implants.

##### 2.2.2.1 Metallic coating

Metallic materials, such as titanium, tantalum and magnesium, have also been used as coatings. Du et al. (Du et al., 2019) investigated the effects of magnesium (Mg) on osteogenesis and bone resorption at a porous structure interface and found that when coated onto implants with porous structures, Mg did not improve osteogenic ability but inhibited peri-implant osteolysis, making it potentially favorable for patients with osteoporosis. Tantalum (Ta) is widely used in the bone repair field due to its high friction factor, nano/microroughness, surface energy, hydrophilicity, etc., and has excellent osteogenic activity, corrosion resistance and antibacterial adhesion (Almeida Alves et al., 2021). To combine the advantages of Ta and PEEK, Zhu et al. (Zhu et al., 2019) added Ta nanoparticles to PEEK and fabricated Ta nanoparticle-reinforced PEEK (Ta-PEEK) materials (with Ta weight ratios ranging from 1% to 9%). *In vitro* and *in vivo* experiments showed that the Ta-reinforced samples, especially 3% Ta-PEEK and 5% Ta-PEEK, enhanced bioactivity and promoted bone formation.

##### 2.2.2.2 Nonmetallic coating

Hydroxyapatite [HA, Ca_10_(PO_4_)_6_(OH)_2_] is the most widely used coating material and has the pure synthetic equivalent components closest to human bone minerals. HA shows osteoconductive ability as well as a remarkable ability to bind directly to bone. Ma et al. (Ma and Guo, 2019) prepared an HA/PEEK biocomposite by incorporating HA particles into the PEEK matrix and reported that the HA/PEEK composites exhibited better adhesion, proliferation and diffusion and higher ALP activity, and the bone integration efficiency around the HA/PEEK composite was higher than that around pure PEEK.

Other carbon materials, such as graphene and its derivatives, have been proven to stimulate cell adhesion, proliferation and migration and have potential for use as scaffolding materials in tissue engineering applications. Graphene oxide (GO), a two-dimensional carbon material, is considered a good bioactive material due to its unique layer-like structure, large specific surface area, sp^2^ carbon domains and abundant oxygen-containing surface functional groups (hydroxyl, carboxyl, etc.) (Kiew et al., 2016). GO-wrapped carbon fiber-reinforced PEEK (CF/PEEK) showed remarkable surface changes. These surface properties, including improved microroughness and nanostructure, had significant positive effects on the initial adhesion, cell proliferation, ALP activity and mineralization potential of bone marrow mesenchymal stem cells (BMMSCs). *In vivo* animal experiments showed that the GO-modified material effectively promoted new bone formation (Qin et al., 2020). Reduced graphene oxide (rGO) is obtained by reducing the oxygen content of GO to a lower level by chemical, thermal or other methods. The structural defects of rGO, in contrast to those of GO, make it easier to interact with biomolecules, cells and polymers. Coating PEEK with rGO can effectively promote osteogenic gene expression *in vitro* and results in good osteogenic properties (Oladapo et al., 2022).

Inorganic ceramic materials have good mechanical strength, surface roughness, and the ability to regulate biological activity by modulating anions and cations. Silicon nitride (SN, Si_3_N_4_) ceramic has the advantages of high mechanical strength, thermal stability and excellent corrosion resistance, while silicon and nitrogen are key components that enhance bone activity and promote bone regeneration. SN has good biocompatibility and bioactivity and exhibits good bone affinity and therefore has received attention as a promising material for skeletal prosthetic implants (Pezzotti et al., 2018). Dai et al. (Dai et al., 2020) demonstrated that SN-coated PEEK presents a higher surface roughness, hydrophilicity, and protein absorption. In addition, SN-coated PEEK slowly released Si ions into simulated body fluid (SBF), resulting in a weak alkaline microenvironment, improving the *in vitro* adhesion, proliferation, differentiation, and gene expression of mouse embryonic osteogenic precursor cells and obviously promoting bone regeneration and osseointegration *in vivo* compared to PEEK. Similarly, Ma et al. (Ma et al., 2020) showed that nanosized calcium silicate (CS, CaSiO_3_)-incorporated PEEK biocomposite (CS/PEEK) exhibited enhanced osteoblast functions *in vitro* and osteointegration *in vivo*. In addition, to compare the bioactivities of Ta and SN, Hu et al. (Hu et al., 2022) fabricated a Ta/PEEK composite (TPC) and SN/PEEK composite (SPC) and showed that TPC significantly stimulated the cell response *in vitro* and promoted new bone regeneration as well as osseointegration *in vivo* compared with SPC. However, the antibacterial property of TPC is lower than that of SPC.

#### 2.2.3 Organic composition coating

Various bioactive organic compositions are used as modification layers, among which polydopamine (PDA) is a mussel-inspired polymer formed by dopamine oxidation and has been considered a good organic surface modification material for PEEK (Liebscher et al., 2013). To determine the direct influence of PDA on cell adhesion, proliferation, and differentiation, Wang et al. (Wang H. et al., 2019) fabricated PDA-coated PEEK and reported that the enhanced hydrophilicity of the PDA-coated PEEK surface facilitated functional protein adsorption and cellular responses, such as enhanced focal cell adhesion, proliferation, and osteogenic differentiation *in vitro*. In addition, the focal adhesion kinase (FAK)/P38 pathways are involved in enhancing the effect of PDA coating on the osteogenic differentiation of BMSCs. Dopamine can form a uniform PDA coating enriched with catechol, amine, imine and quinone during self-polymerization in an alkaline environment. The abundant functional groups can form strong bonds with biomolecules (e.g., peptides), nano-HA and metal ion chelates (e.g., silver ions) (Dong et al., 2020). For example, chelated [Ag(NH_3_)_2_]^+^ ions were reduced to Ag nanoparticles by catechol and deposited on the PDA-coated PEEK surface to prepare PEEK-PDA-Ag. In a rat model of lateral femoral condyle defects, PEEK-PDA-Ag effectively inhibited the growth of injected bacterial cells, and good osseointegration was observed around the PEEK-PDA-Ag implants, indicating that PEEK-PDA-Ag implants have excellent antibacterial ability without affecting osseointegration (Gao et al., 2017).

As a method for obtaining a bioactive surface while maintaining the mechanical properties of implants, LbL self-assembly technology has been widely employed to prepare multilayers on substrate surfaces for biomedical applications. Liu et al. (Liu et al., 2017) fabricated a group of polystyrene sulfonate (PSS) and polyallylamine hydrochloride (PAH) multilayers by LbL self-assembly to obtain bioactivated PEEK. The 20-layer PEEK showed more hydrophilic features than native PEEK, and the surface contact angle was reduced from 39.7 to 21.7°. *In vitro*, modified PEEK resulted in good adhesion and proliferation of bone marrow stromal cells and induced an increased cell growth rate and ALP levels.

**FIGURE 2:**
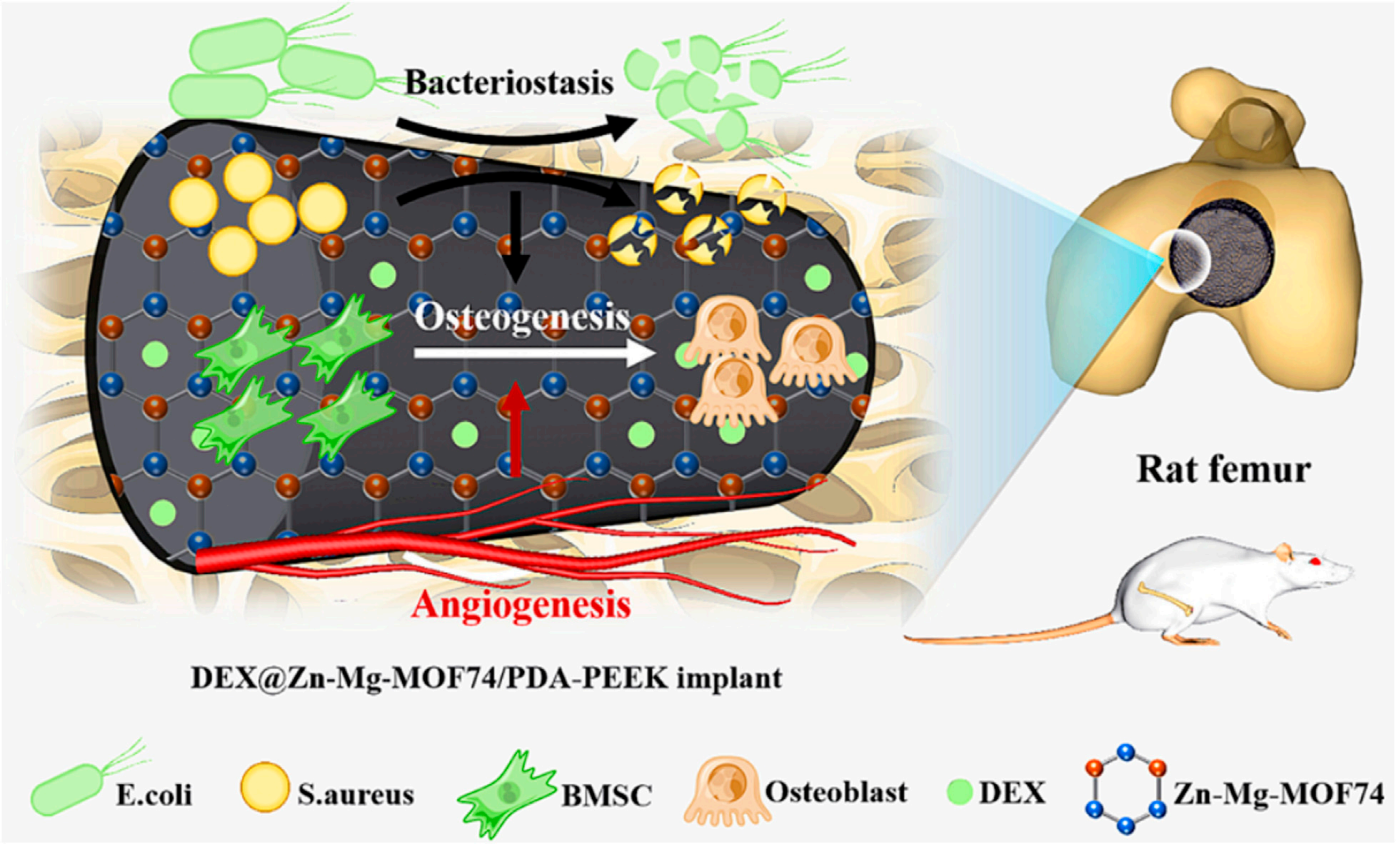
The modification procedures for DEX loaded on the Zn–Mg-MOF74 coating-modified PEEK implant surface and its biological functions: bacteriostasis, angiogenesis, and osteogenesis. Reprinted with permission from Ref. (Dong et al., 2020). 2021, American Chemical Society.

Metal-organic frameworks (MOFs) are coordination compounds formed by metals and organic ligands that have a regular pore structure, an extremely high porosity, a large specific surface area, many functional groups and a low toxicity (Horcajada et al., 2012). Considering that Zn^2+^ can enhance the coating stability, both Mg^2+^ and Zn^2+^ are beneficial for osteogenic activity, and the 3D porous structure of MOFs facilitates cell adhesion, migration, growth and nutrient and metabolite transportation. A Zn-Mg-MOF74 framework organic loaded with dexamethasone (DEX) was constructed on the surface of PEEK by Xiao et al. (Xiao et al., 2021). The results fully demonstrated the beneficial effects of the multifunctional Zn-Mg-MOF74 coating on the bacteriostatic, angiogenesis and bone regeneration properties of implants (Figure 2).

#### 2.2.4 Functional group grafting

Functional group grafting is another method for improving the osteogenic ability of PEEK materials. To study the effect of surface chemistry on protein adsorption and osseointegration, Buck et al. (Buck et al., 2022) attached two different functional groups (-NH_2_ and -COOH) that promoted pro- and anti-inflammatory macrophage responses, respectively, to the surface of sandblasted PEEK with similar wettability and roughness/morphology. The results showed that the -NH_2_ groups promoted mineralization in SBF, and the corresponding surfaces adsorbed more proteins associated with proinflammatory responses, such as Toll receptor signaling. In contrast, the -COOH groups did not promote mineralization in SBF but adsorbed more proteins associated with integrin signaling. Through altered protein adsorption, the surfaces induced different macrophage responses, thus affecting the osteogenic differentiation of mesenchymal stem cells (MSCs). Both mineral binding and the immune response play roles in osseointegration, and the integration of PEEK implants may be improved by mixing these two functional groups to take advantage of their abilities to reduce inflammation and strongly bind bone.

The novel polysaccharide complex strontium Eucommia ulmoides polysaccharides (EUP-Sr) has been proven to exhibit superior bioactivities, including bone immunity regulation and osteogenesis promotion. EUP-Sr grafting on PEEK by the PDA adhesion technique was used to fabricate bioactive PEEK (DPEEK@EUP-Sr). The quantitative reverse transcription PCR (RT‒qPCR) results showed that DPEEK@EUP-Sr downregulated the expression of inflammatory factor-related genes (IL-1β, IL-18 and MMP-9) and upregulated the expression of osteogenic genes (RUNX2 and Col1-α1). Cellular experiments confirmed that DPEEK@EUP-Sr promoted chondrocyte proliferation and enhanced cell adhesion (Mengdi et al., 2022).

Phosphonate group grafting via diazonium chemistry was used to enhance the bioactivity of PEEK. After phosphorylation, MC3T3-E1 cell viability, metabolic activity and calcium-containing mineral deposition were significantly enhanced. Three months after implantation in a critical size cranial defect model, fibrous capsules were visible around the untreated PEEK, whereas they were not seen around the phosphorylated PEEK, and mineral deposition was observed between the phosphorylated PEEK implants and the underlying bone (Mahjoubi et al., 2017). Zheng et al. (Zheng et al., 2019) also successfully introduced phosphate groups onto the PEEK surface by a single-step UV-initiated grafting polymerization technology. *In vivo* evaluations indicated that surface-phosphorylated PEEK exhibited improved bone-implant contact and that poly (vinylphosphonic acid) had excellent bone tissue compatibility. Sulfonation and nitrification treatment are other commonly used PEEK surface modification techniques that can introduce -SO_3_H and -NO_2_ functional groups to the PEEK surface and enhance the bioactivity of PEEK (Li et al., 2019).

## 3 Strategies for improving antibacterial properties

Surgical infection is a common complication in bone tissue engineering and orthopedics, leading to early implant failure. For example, *Staphylococcus aureus*, especially methicillin-resistant *S. aureus* (MRSA), leads to significant morbidity and tissue damage (Qu et al., 2020), which can be a risk factor in bone repair. Thus, the antibacterial properties of PEEK are receiving more attention.

### 3.1 Biomolecule modification

Antibiotics, antimicrobial peptides and natural antibacterial compounds are often used in clinical antibacterial treatments and have been applied in many studies.

#### 3.1.1 Antibiotics

**FIGURE 3:**
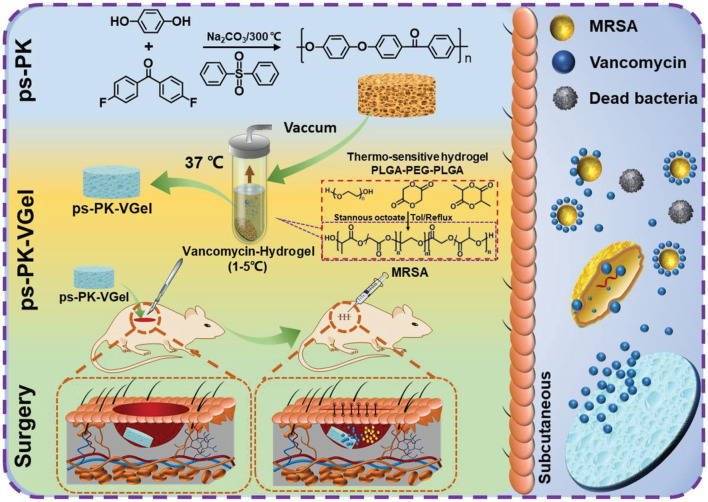
Schematic illustration of the effect and mechanism of porous polyether ketone scaffold-loaded antibacterial hydrogels. Reprinted with permission from Ref. (Mengdi et al., 2022). 2022, Wiley-VCH GmbH.

To slowly release the antibiotic, polylactic-*co*-galactic acid (PLGA)-polyethylene glycol (PEG)-PLGA thermoresponsive hydrogels loaded with vancomycin were designed and used by Qi et al. to fill a high-strength porous PEEK scaffold (Qi et al., 2022). The antibacterial experiments carried out in rats showed effective antibacterial activity, which reduced the infection caused by MRSA and improved the success rate during bone defect repair surgery (Figure 3).

Sun et al. (Sun et al., 2021) obtained PDA and gentamicin sulfate (GS) layer-modified SPEEK (SPEEK–PDA–GS). The self-polymerization of dopamine was performed on H2SO4-treated PEEK (SPEEK) in a solution of dopamine and GS. Xue et al. (Xue et al., 2020) developed an LbL deposition method to rapidly construct brushite (CaHPO4·2H2O) (CaP) layers containing GS on PEEK. CaP- and GS-modified PEEK showed significant antibacterial benefits both *in vivo* and *in vitro*. Yin et al. (Yin et al., 2020) loaded tobramycin (TOB) on a novel multifunctional implant that mainly consisted of 2D titanium carbide (MXene, Ti_3_C_2_T_x_) nanosheets, gelatin methacrylate (GelMA) hydrogels, and bioinert SPEEK. The multifunctional implants displayed robust antibacterial properties against both gram-negative and gram-positive bacteria.

#### 3.1.2 Antimicrobial peptides (AMPs)

**FIGURE 4:**
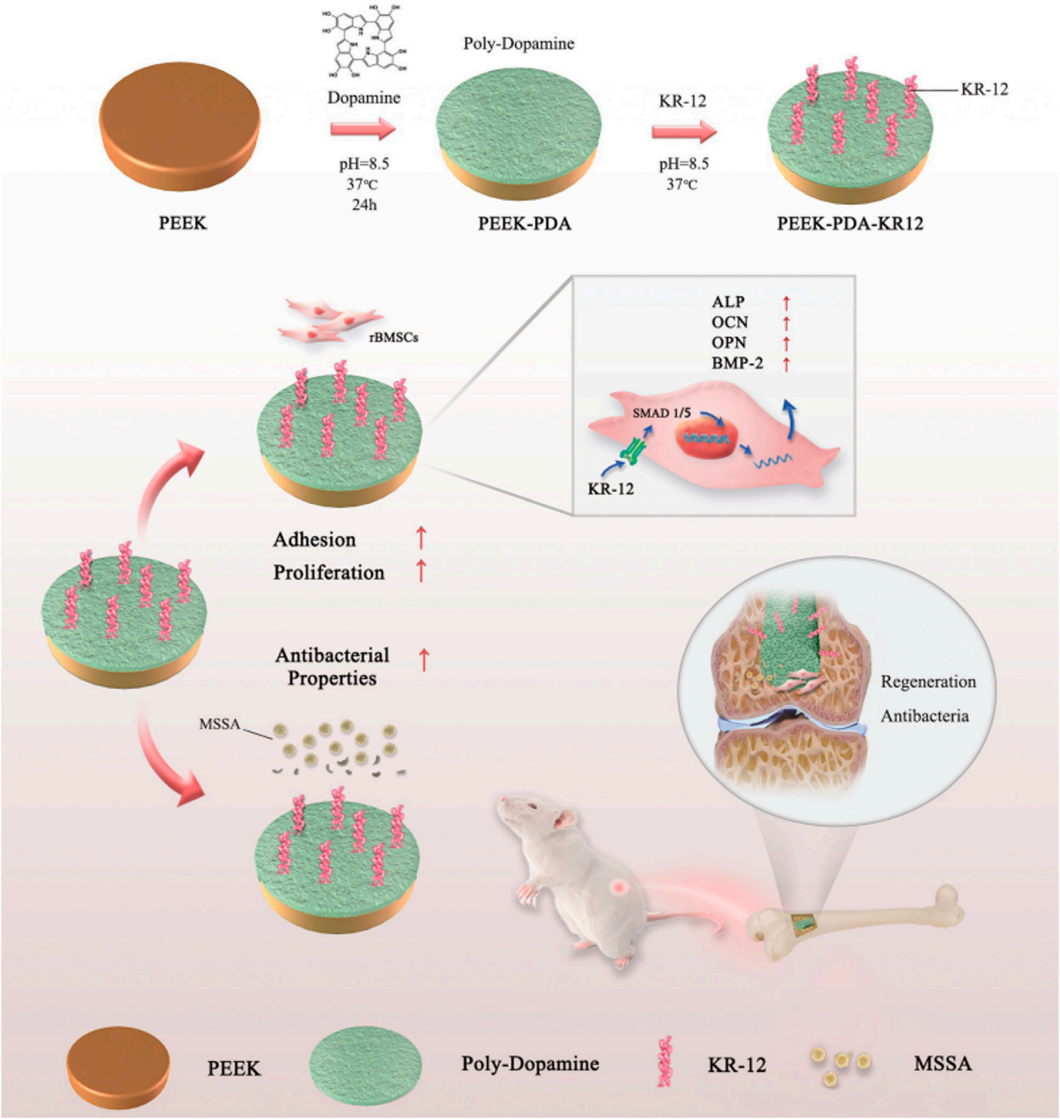
Schematic diagram of the grafting of KR-12 on the surface of PEEK material and its mechanism. Reprinted with permission from Ref. (Qi et al., 2022). 2020, The Royal Society of Chemistry.

In addition to antibiotics, AMPs also show potent antimicrobial abilities. Their good water solubility, thermal stability, tissue nontoxicity and broad spectrum of activity make AMP implants more competitive. KR-12 (residues 18–29: KRIVQRIKDFLR-NH2), the smallest peptide of LL-37, possesses antimicrobial activity. Meng et al. (Meng et al., 2020) immobilized KR-12 on PEEK implant surfaces with the assistance of PDA, which resulted in effective antibacterial activity against *S. aureus* both *in vitro* and *in vivo*. In addition, KR-12 promoted the osteogenic differentiation of BMMSCs through the bone morphogenetic protein/SMAD signaling pathway without cytotoxicity (Figure 4). Yuan et al. (Yuan et al., 2019) immobilized recombinant mouse beta-defensin-14 (MBD-14), a kind of AMP, on a PEEK surface with a 3D porous structure, which resulted in superior antibacterial activity against both *S. aureus* and *Pseudomonas aeruginosa*.

#### 3.1.3 Natural antibacterial compounds

Hinoitiol (HK) is a natural compound from Chamaecyparis obtusa var. formosana with anticancer, antibacterial, anti-inflammatory, and insecticidal activities. It can also lower RANKL-induced osteoclast formation and bone resorption (Wang et al., 2021). Zhang et al. (Zhang et al., 2018) designed hinokitiol-loaded nanobioglass and PEEK composite (mBPC) implants that showed effective antibacterial activity against *S. aureus*. The implants also enhanced osteogenesis *in vivo*.

Genistein, a kind of isoflavone, is a small molecule of phytoestrogen extracted from soybeans and red clover with several biofunctions, such as antioxidant, anti-inflammatory, antimicrobial and antitumor functions. In addition, it enhances osteoblast activity and promotes new bone regeneration through BMP-dependent SMADs and RUNX2 signaling (Dai et al., 2013). Luo et al. (Luo et al., 2022) loaded genistein into the microporous surface of a concentrated sulfuric acid-treated Ta/PEEK composite (TPSG), resulting in antibacterial and osteogenic activity.

Sodium butyrate (SB), a fermentation product of gut microbiota, is involved in various biological processes and shows antimicrobial, anti-inflammatory and immunomodulatory effects. Yang et al. (Yang et al., 2019) loaded SB onto 3D porous SPEEK. Both *in vitro* and *in vivo*, SB-loaded SPEEK, especially with high SB concentrations, exhibited excellent antibacterial activity by augmenting the phagocytic activities of macrophages via reactive oxygen species (ROS).

### 3.2 Inorganic antimicrobial agents

Metal ions such as copper, zinc, selenium and silver can induce good antibacterial properties in close proximity to the surface and can be grafted onto or incorporated into biomaterial surfaces (Rigo et al., 2018). In addition, metal nanoparticles and metallic oxide nanoparticles can also actively kill microorganisms (Guo et al., 2021). While a high metal ion concentration is cytotoxic, the release rate should be balanced in terms of antibacterial activity and nontoxicity.

Copper possesses antibacterial, osteogenic, angiogenic and tissue-healing abilities (Yu et al., 2018). However, the fast release of Cu^2+^ will generate ROS and damage DNA. Liu et al. (Liu et al., 2019) designed a porous microstructure on SPEEK, which can cause bacteria to be trapped. Cu nanoparticles were immobilized on the SPEEK surface to kill the trapped bacteria and control the Cu^2+^ release rate according to the loading amounts. The implants showed superior germicidal activity against MRSA both *in vitro* and *in vivo*.

Silver nanoparticles (AgNPs) also exhibit potential for broad-spectrum antibacterial efficacy activated by bacteria-triggered pH reduction (Li et al., 2020). Deng et al. (Deng et al., 2020) used 3D printing to construct hierarchical porous PEEK scaffolds. AgNPs trapped on the PDA layer formed a unique “pDA-Ag-pDA” sandwich-structured coating, which released Ag + ions triggered by a decreasing pH value. The multifunctional PEEK scaffolds showed satisfactory therapeutic effects against bacteria *in vivo*.

Bimetallic nanoparticles combined with copper and silver displayed enhanced antimicrobial efficacy because of their synergetic effect (Chen X. et al., 2017). In addition to the increased bactericidal capacity, osteogenesis and osseointegration ability were also endowed to implants. Yan et al. (Yan et al., 2020) constructed copper oxide microspheres (μCuO) and AgNPs on a porous PEEK surface through silk fibroin. In a low pH environment *in vitro*, this material released high doses of Cu^2+^ and Ag^+^, killing 99.99% of planktonic bacteria and all sessile bacteria. Promoted bone regeneration and osseointegration induced by these modified implants was observed *in vivo*.

Zinc is essential for normal immune homeostasis and function (Hojyo and Fukada, 2016). Liu et al. (Liu et al., 2018) investigated the immunomodulatory capability of zinc ions by loading a layer of zinc ions on SPEEK biomaterials via a customized magnetron sputtering technique and found that Zn-coated SPEEK can modulate macrophage polarization from a nonactivated to an anti-inflammatory phenotype and induce the secretion of osteogenic and anti-inflammatory cytokines.

### 3.3 Organic antimicrobial agents

Schiff base groups with antibacterial activities can be obtained by chemical reaction on the surface of PEEK. Ding et al. (Ding et al., 2020) treated PEEK with mixed acids of nitric acid and concentrated sulfuric acid to create a multilevel porous structure and then grafted ethylenediamine to achieve amination between the amino groups of ethylene diamine and the ketone carbonyl groups contained in PEEK. This approach provided both Schiff base groups and amino groups that had antibacterial activities.

### 3.4 Synergistic photothermal therapy

**FIGURE 5:**
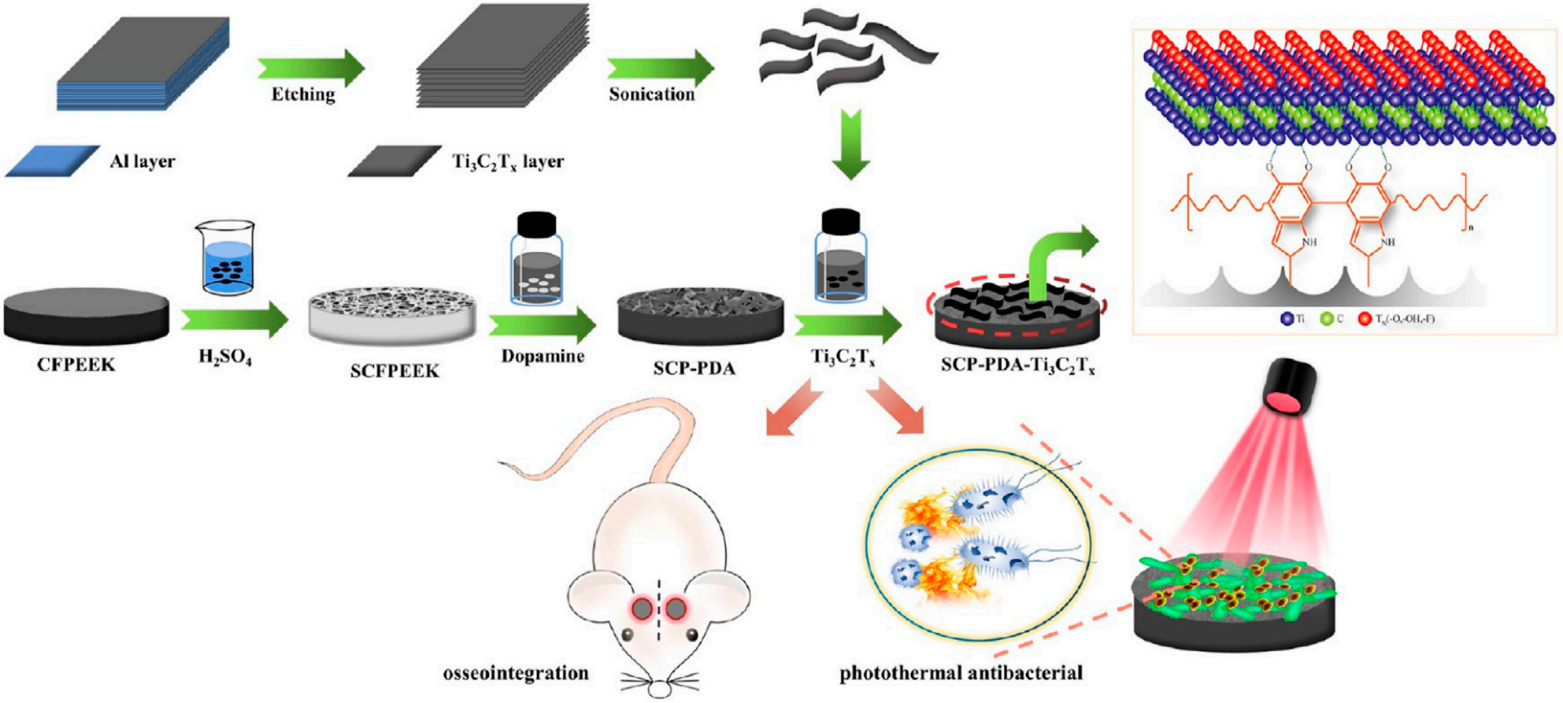
Schematic diagram of the construction of multifunctional orthopedic implant SCFPEEK-PDA-Ti_3_C_2_T_x_. Reprinted with permission from Ref. (Chen X. et al., 2017). 2022, American Chemical Society.

Photothermal sterilization provides a new idea for PEEK modification. Yu et al. (Du et al., 2022) constructed composites with Ti_3_C_2_T_x_ nanosheets, PDA, and sulfonated CFPEEK. During the synergistic photothermal therapy (PTT) treatment of Ti_3_C_2_T_x_/PDA, SCP-PDA-Ti absorbed heat, and the temperature increased to 40.8–59.6°C, which led to bacterial apoptosis (Figure 5).

## 4 Application of PEEK implants in spine surgery

The osteointegration and antibacterial properties of PEEK materials have been improved during these years to display better properties in bone and orthopedics surgery. Meanwhile, many studies have confirmed that PEEK is a potential material compared with traditional materials in spine surgery, trauma repair and joint surgery.

### 4.1 PEEK in lumbar spine surgery

Spinal fusion cages used in spine surgery allow bone growth and fusion of two vertebrae. Three main kinds of material, including titanium and its alloys, PEEK and carbon fiber-PEEK, are widely used in spinal fusion cages.

#### 4.1.1 Traditional PEEK in lumbar spinal surgery

For patients suffering from lumbar disc herniation, lumbar spinal stenosis, or degenerative spondylolisthesis, posterior lumbar hybrid surgery is a treatment option. Titanium rods, which have been traditionally used in this surgery, have sufficient stability and a high fusion rate. As the elastic modulus of titanium alloy is 106–155 GPa, which is too large compared with the elastic modulus of bone, stress shielding, altered loading, and detrimental periprosthetic bone remodeling may occur after surgery (Hak et al., 2014). PEEK rods have been used in lumbar spine surgery since 2007 with good biocompatibility, improved stability, nontoxicity, and an elastic modulus similar to that of bone (Highsmith et al., 2007). The elastic modulus of PEEK is approximately 3.2 GPa, which is between that of cancellous bone (1 GPa) and cortical bone (12–20 GPa) (Golish and Mihalko, 2011). In addition, PEEK rods are transparent in X-ray fluoroscopy and show a small range of artifacts during CT and MRI examinations.

Zhao et al. (Zhao et al., 2022) included 28 patients treated with lumbar posterior hybrid surgery using PEEK rods in a 2-year study and reported that the fusion rate was 100%, and the pain and function were significantly improved. Mechanical complications such as rod breakage or screw loosening did not occur. Adjacent segment disease (ASD), a common complication in lumbar short-segment fusion, was not found. Therefore, considering that the risk of ASD is as high as 75% when using titanium rods for fusion, PEEK rods can be a good choice for hybrid surgery to reduce the risk of ASD complications. In addition, Ogrenci et al. (Ogrenci et al., 2019) evaluated the mid-term radiological and clinical results of 172 patients who underwent PEEK rod operation due to degenerative lumbar spinal diseases. The study confirmed that stabilization with PEEK rods is a safe treatment for degenerative spinal diseases, with low rates of revision and ASD over 2 years. Gao et al. (Gao et al., 2020) confirmed that percutaneous endoscopic lumbar discectomy (PELD) combined with a PEEK rod had good long-term efficacy in symptomatic patients with GLDH (herniation affecting 50% of the sagittal diameter of the spinal canal) through a 2-year trial involving 243 patients. The numerical rating scale (NRS) for back and leg pain and the Oswestry Disability Index (ODI) scores of patients in the PELD + PEEK group were better than those of patients in the PELD group at the final follow-up.

However, Campbell et al. (Campbell et al., 2020) compared 113 patients undergoing lateral lumbar interbody fusion (LLIF) receiving PEEK cages or Ti cages for degenerative indications and found more interbody subsidence into the endplates in the PEEK cage group than in the Ti cage group.

#### 4.1.2 Ti-coated PEEK in lumbar spinal surgery

Subsidence is an undesirable complication of LLIF that may result in a decrease in segmental stability and loss of disc space height and foraminal height. These may ultimately lead to the recurrence of symptoms or additional surgical procedures. Adl Amini et al. (Adl Amini et al., 2021) compared the early subsidence rate of novel 3D-printed Ti *versus* PEEK interbody cages at 6–12 months post-LLIF in 51 female and 62 male patients. The results revealed that the subsidence rate for Grades I-III was significantly lower in the Ti group than in the PEEK group. For high-grade subsidence (Grade II or III), 3D-printed Ti cages had a significantly lower subsidence rate (3.0%) than PEEK cages (18.5%), which suggests that Ti cages decrease the risk of severe subsidence compared with PEEK cages.

Hasegawa et al. (Hasegawa et al., 2020) investigated the interbody bone fusion rates 6 months after posterior lumbar interbody fusion (PLIF) surgery in 149 patients using Ti-coated PEEK (TiPEEK) or PEEK cages and found that the bone fusion rates were significantly higher in the TiPEEK group than in the PEEK group. In addition, the long-term outcomes should also be assessed for a complete evaluation. Schnake et al. (Schnake et al., 2021) found no significant differences in the bone fusion rates between TiPEEK cages and uncoated PEEK cages at 12 and 24 months post-PLIF surgery in a prospective randomized single-center study. This result suggested that both cages can be regarded as equivalent in clinical and radiological results for long-term outcomes. Zhu et al. (Zhu et al., 2021) evaluated the radiographic and clinical outcomes of 64 patients who underwent transforaminal lumbar interbody fusion (TLIF) using PEEK cages with titanium (Ti) and hydroxyapatite (HA) coatings (PEEK-Ti-HA cages) or uncoated PEEK cages for 2 years. The PEEK-Ti-HA cages, in contrast with the uncoated PEEK cages, produced a better fusion rate at 3 months after single-level TLIF. The fusion rates of both groups reached 100% at the final follow-up.

### 4.2 PEEK in cervical spine surgery

Anterior cervical discectomy and fusion (ACDF) is a standard neurosurgical treatment for degenerative diseases of cervical intervertebral discs. The assessment of PEEK cages used in single- and multilevel ACDF was reported by Zapolska et al., including 30 patients with single- or multilevel cervical disc herniation. The one-year clinical and radiological evaluation suggested complete fusions in all patients and a significant reduction in pain severity in 97% of patients. No evidence of secondary ASD was found 1 year postsurgery (Zapolska et al., 2019). It was reported that both Ti and PEEK cages showed a 100% fusion rate at 1 year post-ACDF in a trial including 149 patients. No significant difference in clinical and radiological outcomes was found between the two groups (Junaid et al., 2018). Godlewski et al. (Godlewski et al., 2022) reported the use of PEEK cages or TiPEEK cages in 104 patients undergoing ACDF. Complete fusion was observed in 88.2% of disc spaces for PEEK cages compared to 44.1% for TC-PEEK cages at 12 months post-ACDF.

However, Krause et al. (Krause et al., 2018) demonstrated that PEEK implants lead to a fivefold higher rate of pseudarthrosis than allografts in 1-level ACDF. A total of 52% of the 56 patients with PEEK implants displayed radiographic evidence of pseudarthrosis, compared to 10% of the 71 patients with structural allografts. In addition, seven patients with PEEK implants required reoperation for pseudarthrosis, compared to 1 patient with an allograft. This result should be considered before performing 1-level ACDF. Ryu et al. (Ryu et al., 2021) compared the radiographic and clinical outcomes of 194 patients undergoing 1-3 level ACDF with PEEK or allograft implants (79% allograft *versus* 21% PEEK). Thirty-nine percent of the patients with PEEK implants exhibited radiographic nonunion, which was significantly higher than the 27% of patients with allograft implants that exhibited the same. In addition, they found that multilevel procedures, younger age, and active smoking status might lead to radiographic nonunion. These factors can be controlled during surgery and provide insight for choosing implants and surgical plans.

**FIGURE 6:**
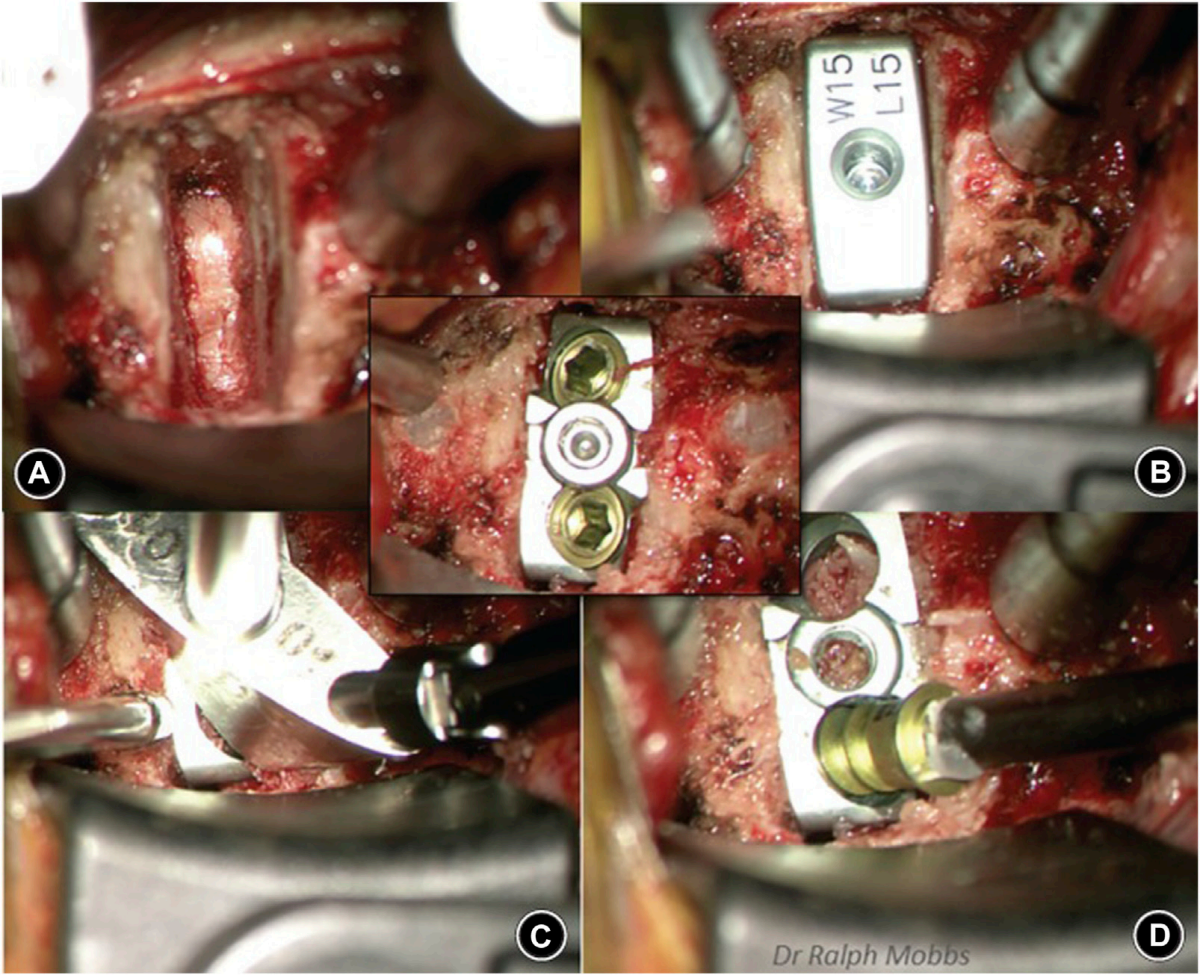
Sequence of anterior cervical discectomy and fusion (ACDF) using the ACDF device. Reprinted with permission from Ref. (Junaid et al., 2018). 2019, Chinese Orthopedic Association and John Wiley & Sons Australia, Ltd.

In addition, Phan et al. (Phan et al., 2019) reported the early clinical and radiological outcomes of using a uniquely designed integral fixation Ti/PEEK cage packed with supercritical CO_2_ sterilized allograft in ACDF. Data from 47 consecutive patients who were followed up for a minimum of 6 months showed a 96% fusion rate and no cases of implant Ti/PEEK delamination or implant failure, which suggests the superior early integration of the implants with the surrounding bone and vertebral endplate (Figure 6).

## 5 Application of PEEK in joint surgery

Due to the high mechanical strength, good stability and X-ray permeability of PEEK material, it can be used in joint repair and arthroplasty.

### 5.1 PEEK in suture anchor

Suture anchors are necessary in rotator cuff repair and are made of different materials, including metal, polyglycolic acid (PGA), and polylactic acid enantiomers (PLLA). The first-generation suture anchor, metallic anchors, leads to anchor loosening and migration after the operations (Ozbaydar et al., 2007). Although biodegradable anchors can increase bone generation and avoid signal artifacts on MRI in postoperative assessments, complications such as osteolysis, chondral injury, synovitis and arthropathy also appear due to the degradation of polymers (Dhawan et al., 2012). Compared with those anchors, PEEK anchors with newer composites display lower complications and degradation after surgeries.

In a prospective randomized trial, Kim et al. compared the clinical outcomes of open-construct PEEK suture anchors with nonvented biocomposite anchors in arthroscopic rotator cuff repair and found that better bone ingrowth was provided by open-construct PEEK anchors at 6 months after surgery than by nonvented biocomposite anchors, which suggested that open-construct PEEK anchors led to more effective early rotator cuff healing than nonvented anchors (Kim et al., 2020).

However, Kim et al. (Kim et al., 2019) indicated that PEEK anchors showed an unexpectedly high rate of fluid formation (41.7%) in a study that aimed to compare the osseous reactions elicited by all-suture, polyetheretherketone (PEEK), and biodegradable anchors after rotator cuff repair. This postoperative biological reaction should be considered before surgery.

In addition, PEEK interference screws are used in anterior cruciate ligament (ACL) reconstruction. Shumborski et al. (Shumborski et al., 2019) proved that PEEK interference screws show similar clinical performance to titanium interference screws in ACL reconstruction in 2 years. They evaluated the graft rerupture rate, contralateral ACL rupture rate, subjective outcomes, and objective outcomes, and there were no significant differences. The absence of metal artifacts ensures the assessment of PEEK interference screws.

Christensen et al. (Christensen et al., 2018) applied a PEEK bullet-in-sheath tenodesis device in tendon transfers. In bone analog models and porcine bone models, the PEEK sheath-based interference device showed superior tendon pullout strength and maintained the integrity of the tendon graft, which may provide an option for tissue repair in the foot and ankle.

### 5.2 PEEK in arthroplasty

Knee arthroplasty is a treatment for knee osteoarthritis that can restore joint function and relieve patient pain. Metal components have been used as artificial knee joints, while PEEK materials are considered the new generation of keen replacements. Du et al. (Du et al., 2018) recorded the safety and performance of a cemented PEEK-based knee replacement device in a goat model and found the promising use of PEEK-on-PE knee replacement, which is feasible and safe in surgical intervention. In addition, PEEK insert liners are also used in total hip arthroplasties. Heijnens et al. (Heijnens et al., 2021) evaluated the outcomes of carbon fiber-reinforced PEEK (CFR-PEEK) insert liners used as bearings in cementless total hip arthroplasties over 14.3 years of follow-up. Although the superior mechanical and stable chemical behavior of CFR-PEEK has been reported, CFR-PEEK inserts displayed an obviously lower survival rate than ultrahigh molecular weight polyethylene pipe (UHMWPE), proving a disappointing long-term survival rate.

### 5.3 PEEK in high tibial osteotomy (HTO)

HTO is a well-established treatment for medial compartment cartilage defects, osteoarthritis, and varus knee malalignment. Medal plates and PEEK plates are involved in the fixation system, which resist the high loading of early full weight-bearing programs and improve the clinical outcomes. Hartz et al. (Hartz et al., 2019) retrospectively analyzed the outcomes of CFR-PEEK plate-related open wedge HTO and found that open wedge HTO using a CFR-PEEK plate for patients leads to outstanding bony consolidation in cases with a hinge fracture and a gap size >12 mm as well as for severely obese patients. Hevesi et al. (Hevesi et al., 2019; Keyt et al., 2022) compared the outcomes of metal and PEEK implants used in opening-wedge HTO and assessed the varus deformity correction, arthroplasty rates and short-to mid-term hardware removal. They found that both metal and PEEK implants could maintain coronal varus deformity correction effectively. Specifically, 2-year and 5-year hardware removal-free survival for PEEK was significantly superior to that for metal.

## 6 Application of PEEK in trauma repair

Cranioplasty and fracture fixation are the main operations in trauma repair. The material choices for operations are improving constantly in recent years, and different materials have their own strengths and weaknesses.

### 6.1 PEEK in cranioplasty

Titanium, autologous bone and other materials have been used in cranioplasty, while PEEK implants have recently gained popularity because of their favorable characteristics (Yang et al., 2020). Mozaffari et al. (Mozaffari et al., 2022) compared PEEK cranioplasty with autologous cranioplasty and concluded that PEEK biomaterials may offer a superior complication profile with similar hospital LoS compared to autologous bone implants used in cranioplasty.

**FIGURE 7:**
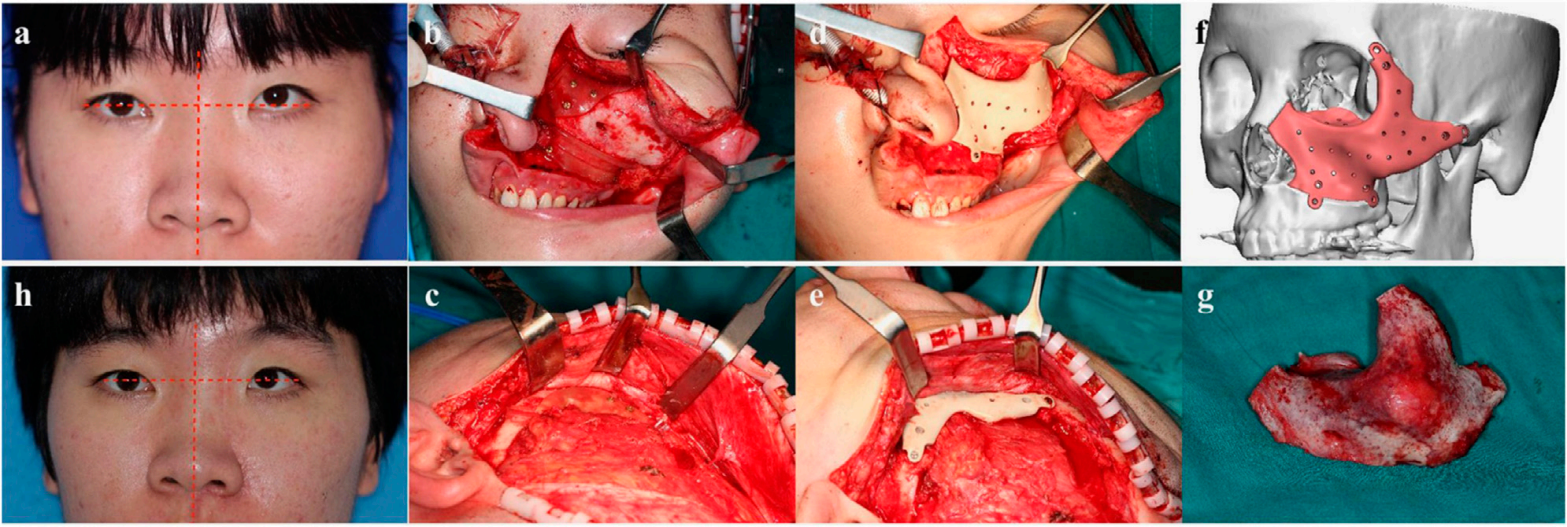
A typical patient with fibrous dysplasia in the left OMZ complex reconstructed with a PEEK PSI implant. Reprinted with permission from Ref. (Hevesi et al., 2019). 2021, Elsevier Ltd.

The orbital–maxillary–zygomatic (OMZ) complex is an important part of the midface, which requires implant shape adaptability and biocompatibility. Lv et al. (Lv et al., 2022) used custom-made patient-specific implants by virtual surgical planning to repair bone defects in OMZ complexes with PEEK. The 12 patients undergoing primary/delayed reconstruction of defects in the OMZ complex were considered to have received successful operations. The postoperative appearance and function, which were assessed after 6 months, showed excellent facial symmetry and globe position. No obvious complications occurred, and patient-specific PEEK implantation showed excellent biocompatibility and clinical outcomes (Figure 7).

### 6.2 PEEK in fracture fixation

CFR-PEEK, which is considered a new material in fracture fixation, has great strength, biocompatibility, resistance and X-ray permeability. Takashima et al. (Takashima et al., 2020) assessed CFR-PEEK implants among 20 patients suffering proximal femoral fractures and found no adverse reactions or failures. No complications, pain, or radiological evidence of failure occurred, which indicated the efficacy and safety of CFR-PEEK intramedullary nails. In addition, they compared CFR-PEEK intramedullary nails with metallic intramedullary nails for the treatment of intertrochanteric femoral fractures in 40 patients. The CFR-PEEK intramedullary nails showed preferable fracture site visibility on radiographs, which provides advantages for fracture reduction and bone formation evaluation (Takashima et al., 2021).

Chloros et al. (Chloros et al., 2022) assessed 361 patients in 9 studies who were treated with CFR-PEEK plates or conventional plates for the fixation of extremity fractures, including proximal humerus fractures, distal radius fractures, distal femur fractures and ankle fractures. The CFP-PEEK plates showed higher union rates and lower complication rates than conventional plates.

## 7 Conclusion and future perspectives

In this review, we summarized the development of PEEK as a biomedical material in bone tissue engineering and orthopedics in recent years. Although PEEK implants have already shown outstanding performance in orthopedics, basic studies on bioactivity can provide better PEEK materials through clinical transformation. PEEK has shown great potential for use in the medical field as an implant material, and its future prospects are bright. Here are some of the ways PEEK is expected to be used in the future:

(1) Improving Osteointegration: Researchers are exploring ways to improve this process by modifying the surface of PEEK implants to promote better bone growth. For example, surface roughening or coating with bioactive materials such as hydroxyapatite can enhance osteointegration.
(2) Antibacterial Properties: Infections are a major concern with any type of implant surgery. Researchers are working to enhance antibacterial properties by adding antimicrobial agents to the material. This could significantly reduce the risk of infection and improve patient outcomes.
(3) Customization: PEEK is easy to shape and can be manufactured into custom implants for individual patients. This allows for a more precise fit and better outcomes. In the future, 3D printing technology may be used to create PEEK implants that are even more customized and precise.
(4) Reduced Imaging Artifacts: One of the drawbacks of metal implants is that they can cause imaging artifacts on MRI and CT scans, making it difficult to get clear images of the surrounding tissue. PEEK is radiolucent, which means it does not cause these artifacts (Kurtz and Devine, 2007). This makes it easier for doctors to monitor the healing process and detect any issues that may arise.
(5) Versatility: PEEK can be used in a variety of medical applications, including spine surgery, joint replacement, and trauma repair. As more research is conducted and new techniques are developed, it is likely that PEEK will be used in even more areas of medicine.

With ongoing research and development, we can expect to see even more innovative uses of this versatile material in the years to come.
